# Measuring Use of Resourcefulness Skills: Psychometric Testing of a New Scale

**DOI:** 10.5402/2011/787363

**Published:** 2011-06-07

**Authors:** Jaclene A. Zauszniewski, Abir K. Bekhet

**Affiliations:** ^1^Frances Payne Bolton School of Nursing, Case Western Reserve University, 10900 Euclid Avenue, Cleveland, OH 44106-4904, USA; ^2^Marquette University College of Nursing, Milwaukee, WI 53233, USA

## Abstract

Highly resourceful individuals have been found better able to meet life's challenges and to experience more positive health outcomes. Although psychometrically sound measures of resourcefulness exist and resourcefulness training trials show that the intervention increases adaptive functioning and enhances quality of life, there is no direct measure of intervention fidelity. This study examined the reliability and validity of an 8-item Resourcefulness Skills Scale (RSS), which measures the frequency with which intervention recipients use specific resourcefulness skills. The RSS was found to have acceptable internal consistency (*α* = .78), criterion-related validity (*r*'s = .50 and .52 with other resourcefulness scales), and construct validity (*r*'s =.38 and .53 with theoretically-related constructs). Factor analysis revealed two factors reflecting personal and social resourcefulness. Because the RSS queries respondents on their use of skills taught during resourcefulness training, it has potential usefulness as a measure for evaluating how well the training is translated into use of the skills in daily life.

## 1. Introduction

The middle range theory of resourcefulness [1] is grounded in a conceptualization of two forms of resourcefulness: personal (self-help) and social (help-seeking) resourcefulness. According to the theory, the cognitive-behavioral skills are complementary and equally important for attaining, maintaining, or regaining health despite potentially adverse situations [1, 2]. Persons with greater resourcefulness have been found to be better able to deal with challenging situations in a more adaptive, constructive manner than persons with low resourcefulness and to have better quality of life and greater life satisfaction [3–9]. Furthermore, research has shown that those who are high in both personal and social resourcefulness have better adaptive functioning and less anxiety and depression than those who are high on social or personal resourcefulness alone [1]. 

While the research on resourcefulness has focused on numerous groups, including pregnant women [4], women with diabetes [8], school-aged children [10], elders with chronic conditions [9, 11], and caregivers of elders with dementia [12]. Family members of persons with serious mental illnesses, including schizophrenia, major depressive disorder, bipolar disorder, panic disorder, and others, have received limited attention [13, 14]. Individuals who have a family member diagnosed with a serious mental illness are at risk for poor health and low quality of life because of the stress and stigma by association that they experience [15, 16]. Women typically serve as the primary care providers of persons with serious mental illness [17], and two studies of women family members of adults with serious mental illness have suggested that they may benefit from learning personal and social resourcefulness skills to manage their stress and cope with their daily tasks [16, 18]. 

The self-help skills that constitute personal resourcefulness, also referred to as “learned” resourcefulness, involve strategies for coping independently with daily tasks and the stress that may be involved in completing them [19]. Personal resourcefulness skills include the use of cognitive reframing, positive thinking, problem-solving, priority-setting, and planning ahead. The help-seeking skills that constitute social resourcefulness include seeking help from others when one is unable for any reason to function independently [20]. Formal sources of help might include professionals: medical, legal, financial, and so forth, and community organizations: hospitals, clinics, and so forth. Informal sources include family members and friends. Both sets of skills, personal and social, are believed to be learned through either formal or informal instruction [19] and they can, therefore, be taught [11, 21].

Resourcefulness training consists of teaching personal and social resourcefulness skills by use of an 8-letter acronym that spells the word RESOURCE, which is used to facilitate recall of specific resourcefulness skills: Rely on family/friends; Exchange ideas with others; Seek professional help; Organize daily activities; Use positive self-talk; Reframe the situation positively; Change from usual reaction; Explore new ways. The first three skills include help-seeking behaviors (social resourcefulness) and the last five involve self-help strategies (personal resourcefulness). However, according to resourcefulness theory [1, 19], the skills constituting resourcefulness must be reinforced and practiced, not just learned. Therefore, during resourcefulness training, personal and social resourcefulness skills can be practiced and reinforced through either individualized or group approaches [11, 21].

Several studies have shown that teaching personal and social resourcefulness skills promotes healthy physical, psychological, and social functioning [11, 21]. However, to date, studies of the effectiveness of resourcefulness training have not incorporated measurement of intervention fidelity, that is, whether the intervention was implemented according to a planned protocol [22, 23]. One method to obtain information on fidelity is the use of a reliable and valid measure to evaluate the content of the intervention. For example, in the case of resourcefulness training, measurement of the degree to which intervention recipients report their use of the personal and social resourcefulness skills taught to them would provide useful evaluative information concerning the delivery of the resourcefulness training intervention. There are a number of measures of resourcefulness and similar constructs exist, including the 36-item Self-Control Schedule (SCS) [19], a well-known measure of “learned” (or personal) resourcefulness, and two measures of social resourcefulness: the Social Resourcefulness Scale (SRS) [24] and the Help-Seeking Behavior Scale [25]. However, these measures do not evaluate the use of the specific personal (self-help) and social (help-seeking) skills taught during resourcefulness training.

Another measure of resourcefulness is the Coping Resources Inventory for Stress (CRIS) [26], which appears to capture aspects of both personal and social resourcefulness. However, the CRIS does not specifically evaluate the teaching of resourcefulness skills and its length (280 items) may be burdensome for assessing intervention fidelity within clinical trials. More recently, Zauszniewski and colleagues [2] developed the 28-item Resourcefulness Scale (RS), which reflects both personal resourcefulness and social resourcefulness. However, although the RS captures whether respondents use personal and social resourcefulness skill in specific situations, it does not evaluate the teaching of of specific personal and social resourcefulness skills. Therefore, this study examined the reliability and validity of an 8-item Resourcefulness Skills Scale (RSS) that can be used to measure of the enactment of both personal and social resourcefulness as a first step toward establishing its future usefulness as a measure to evaluate intervention fidelity.

## 2. Materials and Methods

### 2.1. Design

This psychometric study was a secondary analysis of cross-sectional data obtained from face-to-face interviews with 60 women family members of adults diagnosed with a serious mental illness. The interviews were conducted by trained data collectors in the women's homes or other mutually agreed upon, but private venues in the community. Approval from the University Institutional Review Board was obtained prior to data collection. The findings from the parent study [16] and follow-up analyses have been reported elsewhere [18, 27]. 

### 2.2. Sample

Women family members were recruited by flyers advertising the study that were posted in various places in Northeast Ohio communities, including restaurants, supermarkets, salons, department stores, libraries, churches, and physician offices. To be included in the study, the women family members had to be adults (ages 21 through 65 years), able to read and understand the English language, and a member of a family with an adult who had a diagnosed serious mental illness (schizophrenia, bipolar disorder, major depression, or anxiety disorder); however, they did not need to be living in the same household. The sample size of 60 exceeded the minimum requirement of four subjects per item on a scale that is recommended for psychometric analysis [28]. The subject to item ratio for the 8-item RSS was 1 to 7.5.

The 60 women who participated in the study had an average age of 46 years (SD = 11.71; range 23 to 65 years). The parent study involved targeted recruitment of equal groups of African-American and white women. Half of the sample had completed some college or had an associate degree, and most of them (40%) were mothers. Family members had diagnoses of schizophrenia (45%), bipolar disorder (45%), major depression (8%), or panic disorder (2%) [16]. Less than half of the women (40%) reported living in the same household with the family member who had a serious mental illness. Nevertheless, more than two thirds (68%) of the women said that they provided direct care to their mentally ill family member. In this sample, there were no differences in resourcefulness by race/ethnicity or level of education and no differences between those who lived with the mentally ill person and those who lived apart, or between those who provided care/assistance to their mentally ill family member and those who did not [16].

### 2.3. Instruments

In addition to collecting demographic and descriptive information about the women family members, we administered the 8-item Resourcefulness Skills Scale (RSS) during the face-to-face interview to measure the frequency of use of the eight specific skills. Three items measure the frequency of using help-seeking or social resourcefulness skills (relying on family or friends, seeking professional help, and social exchange), and five items address the frequency of using self-help or personal resourcefulness skills (use of positive self-statements, cognitive reframing, exploring ideas, behavioral change, and organization). Respondents are asked how frequently they use each of the eight skills on a 4-point scale ranging from never (0) to always (3). Scores may range from 0 to 24 with higher scores indicating more frequent use of resourcefulness skills.

To establish validity, we administered two other measures of resourcefulness and two measures of theoretically related constructs during the face-to-face interview. The other measures of resourcefulness were Rosenbaum's Self-Control Schedule [19] and Zauszniewski's Resourcefulness Scale [2]. The measures of theoretically related constructs were the Depressive Cognition Scale [29] and the Short Form Health Survey (SF-12) [30]. These four measures are briefly described below.

The 36-item Self-Control Schedule (SCS) [19], a well-known measure of learned resourcefulness, captures the use of self-control skills as well as belief in one's ability to manage adversity [19]. However, seeking help from others is not viewed as resourceful. Respondents use a 6-point Likert scale to indicate the degree to which they believe each item characterizes their behavior, ranging from not at all like me (0) to very much like me (5). Scores may range from 0 to 180; higher composite scores, after reverse scoring for 11 negatively phrased items, indicate greater learned resourcefulness. Internal consistency estimates from  .79 to  .83 have been reported in young through middle-aged women [8, 9, 12, 31]. Construct validity has been demonstrated by significant correlations in the expected directions between the SCS and measures of social support and daily hassles in women caregivers [12], depressive symptoms and acceptance in young to middle-aged women with diabetes [8, 31], and daily functioning in mothers of school-aged children [10]. 

The 28-item Resourcefulness Scale [2] contains items reflecting both personal (self-help) resourcefulness (16 items) and social (help-seeking) resourcefulness (12 items). Respondents indicate the degree to which each item describes their behavior, ranging from extremely nondescriptive (0) to extremely descriptive (5). Scores may range from 0 to 140, with higher scores indicating greater resourcefulness [2]. The scale has demonstrated internal consistency with a Cronbach's alpha of  .85 [2]. Evidence for construct validity was supported by confirmatory factor analysis indicating the presence of the two forms of resourcefulness: the 16 items from the personal resourcefulness subscale loaded cleanly on one factor, while the 12 items from the social resourcefulness subscale loaded cleanly on a second factor; no items had cross-loadings that exceeded  .30; substantial intercorrelations between the two factors further supported the scale's construct validity [2].

The 8-item Depressive Cognition Scale (DCS) [29] was used to measure depressive cognitions, which are believed to be negatively associated with resourcefulness, according to resourcefulness theory [1, 19]. Therefore, higher scores on the RSS would be expected to correlate significantly with lower scores on the DCS. The DCS uses a 6-point Likert scale from strongly agree (5) to strongly disagree (0) to indicate the degree to which a particular statement describes the individual's current thoughts. The items are phrased positively so that strong disagreement with an item indicates the presence of a depressive cognition. Scores may range from 0 to 40, with higher scores, after reverse coding of all 8 items, indicating more depressive cognitions. The DCS has reported internal consistency with Cronbach's alphas ranging from  .75 to  .87 [31, 32]. Construct validity was supported by correlations in the expected directions (*p* < .001) with caregiver burden, resourcefulness, sense of coherence, and quality of life (*r*′s = .40, −.65, −.77, −.70, resp.) [32]. Confirmatory factor analysis produced a single factor with 48% of the variance explained and factor loadings >.45 for all items [32]. 

Quality of life was measured by the Short Form 12-item Survey (SF-12) [30], which contains two subscales reflecting physical function and psychological well-being. Taken together, the two subscales reflect overall quality of life. According to resourcefulness theory [1], persons who are more resourceful experience better quality of life. Therefore, higher scores on the RSS would be expected to correlate significantly with higher scores on the SF-12. The simplified method of scoring [33] was used, and four items were reversed coded to indicate better mental or physical health; scores on all 12 items were then summed to provide a composite estimate of quality of life. Using this scoring method, internal consistency estimates have ranged from  .72 to  .89, and construct validity has been demonstrated by confirmatory factor analysis, contrasted groups, and hypothesis testing [33].

## 3. Results and Discussion

Data were analyzed using the International Business Machines (IBM) Statistical Package for the Social Sciences (SPSS) software version 19.0. Preliminary data analysis included examination of frequency distributions and descriptive statistics (e.g., means, standard deviations, and ranges) to ensure that the statistical assumptions for correlational analysis to test construct validity were not violated. The means, standard deviations, and ranges on measures of learned resourcefulness, resourcefulness, depressive cognitions, and quality of life are shown in Table 1.

### 3.1. Reliability

Cronbach's alpha for the 8-item RSS was  .78, indicating acceptable internal consistency since the suggested minimum for coefficient alpha is  .70 [34]. In addition, there was evidence that the internal consistency of the scale would not be improved if any of its items were deleted.  Table 2 displays the alphas for the total scale if any of the eight items were deleted. 

The internal consistency alpha of  .78 is consistent with findings from studies of other resourcefulness measures. A previous study of the resourcefulness scale (RS) (28 items measuring both personal and social resourcefulness), for example, results reported an alpha of  .85 in a sample of older adults [2]. However, instruments with greater numbers of items are more likely to have large alphas [35].

Item-to-total scale correlations were examined to determine the homogeneity of the RSS (see Table 2), defined as a substantial number of scale items with corrected item-to-total scale correlations between  .30 and  .70 [36]. All 8 RSS items had item-to-total scale correlations within the recommended range, similar to the findings for the 28-item RS in older adults [2]. Reliability of the RSS was also evaluated by examining interitem correlations, which averaged  .31. These correlations are shown in Table 3. Interitem correlations should average between  .30 and  .70; correlations above  .70 imply item redundancy [34]. Applying this criterion, 54% of the 28 possible interitem correlations fell within the desired range. Thirteen interitem correlations fell below the minimum criteria of  .30, and none were indicative of redundancy. The correlations that fell outside the recommended range might have reflected the sample characteristics or composition and, therefore, require cautious interpretation. 

The findings suggest that the RSS is a reliable measure in terms of internal consistency. This is important for measurement of the use of skills captured by the RSS because, collectively, they reflect an individual's resourcefulness [37]. Consistent measurement of these skills taught in resourcefulness training is important for assessing intervention fidelity, defined as the competent delivery of an intervention that adheres to a prescribed protocol [38, 39]. 

### 3.2. Validity

Criterion validity was examined by comparing the RSS to established, psychometrically sound measures of resourcefulness, including the SCS [19] and the RS [2]. Both the SCS [19] and the RS [2] query respondents about their general use of resourcefulness skills in specific situations, but neither provides information about the frequency of use of resourcefulness skills, as the RSS does. Significant positive correlations were found between the RSS and the SCS (*r* = .50; *p* < .001) and the RS (*r* = .52, *p* < .001). Both of the correlation coefficients were in the moderate range [40], possibly indicting that the frequency of use of resourcefulness skills may not perfectly reflect an individual's perception of whether certain resourcefulness skills are characteristic of them.

Construct validity was determined by examining the strength, direction, and significance of the correlations between the RSS and measures of the theoretically related constructs depressive cognitions and quality of life. Significant correlations in the expected directions were found between the RSS and depressive cognitions (*r* = −.53; *p* < .001) and quality of life (*r* = .38, *p* < .003), indicating that more frequent use of resourcefulness skills is associated with fewer depressive cognitions and better quality of life. Although the correlation between resourcefulness skills and quality of life was marginal, the correlation between resourcefulness skills and depressive cognitions was moderate [41]. 

Exploratory factor analysis was used to further examine the construct validity of the RSS in this sample of women family members of adults with serious mental illness. Before conducting the factor analysis, the adequacy of the sample and the data were examined in relation to specific statistical parameters [42]. The KMO index of  .684 indicated sampling adequacy [40], and Bartlett's test of sphericity (*X* ^2^ = 144.34; *p* < .001; determinant = .074) indicated that the correlation matrix was suitable for factor analysis [42]. Having met the conditions of adequate sample size and suitability of the correlation matrix, the data from the 8-item RSS were then subjected to factor analysis using the principal axis factoring method of extraction with oblimin rotation. Oblique rotation was used because a previous factor analytic study of the RS had suggested correlated factors [2]. 

The factor analysis resulted in a solution in which the 8 RSS items loaded on two factors, and 47% of the variance was explained. The 5 items of the RSS that reflected the frequency of use of self-help or personal resourcefulness skills loaded on the first factor, while the 3 items measuring the frequency of use of help-seeking or social resourcefulness skills loaded on the second factor. No items loaded on both scales. However, the two factors were correlated (*r* = .36, *p* < .001), suggesting that they are related but not redundant and may reflect two dimensions of a single construct. 

Factor loadings ranged from  .51 to  .85.  Table 4 gives the factor loadings. The findings suggest that RSS is a valid measure, which is very important in terms of evaluating intervention fidelity.

When using IBM-SPSS, evaluation of how well the factor analytic model fits the data is limited to the chi-square statistic as a test of goodness of fit, and this has been criticized for its sensitivity to sample size and the nature of the data [43]. An alternative index that minimizes the influence of sample size on the chi-square statistic is the relative/normed chi-square, determined by dividing the chi-square value by the degrees of freedom [44]. The recommended acceptable values for this statistic range from 2.0 [45] to 5.0 [44]. In the case of this two factor solution, the chi-square test of goodness of fit indicated that the model fit the data fairly well (*X* ^2^(28) = 144.34, *p* < .001). The relative/normed chi-square value indicated acceptable model fit (144.34/28 = 5.16). Further testing of the RSS with more diverse populations is recommended so that the factor structure can be validated beyond women family members of adults with mental illness.

The findings from the examination of the validity of the RSS suggest that it is a valid measure. That is to say, the RSS is measuring the construct it purports to measure, and it approximates a previously established “gold standard” measure [46]. Establishing the validity of a measure to evaluate intervention fidelity is of critical importance [47]. Although it is recommended that the RSS be tested further in more diverse populations, for women family members of persons with mental illness, the RSS provides an intervention-specific measure for evaluating the personal and social resourcefulness skills taught during resourcefulness training.

## 4. Conclusion

This study examined the reliability and validity of the newly developed 8-item Resourcefulness Skills Scale. The scale differs from other measures of resourcefulness: it is shorter and focuses on measurement of the frequency of use of the specific self-help (personal resourcefulness) and help-seeking (social resourcefulness) skills taught during resourcefulness training. Personal resourcefulness skills include using positive self-talk, cognitive restructuring, positive behavior change, problem-solving, and organization. Social resourcefulness skills include reliance on family and friends, interpersonal exchange, and seeking of professional help or expert advice. 

This analysis of the psychometric properties of the RSS in women family caregivers of persons with serious mental illness yielded promising evidence of the scale's reliability and validity and evidence of the presence of both personal and social dimensions of resourcefulness, which is consistent with Resourcefulness Theory [1]. Thus, the RS effectively evaluates the frequency of use of the 8 specific resourcefulness skills that reflect personal and social resourcefulness, which is important for evaluating the fidelity of resourcefulness training. Resourcefulness training can be done in clinical and community settings, and it has been found to be effective in pregnant women [4], women with diabetes [8], school-aged children [10], elders with chronic conditions [9, 11], and caregivers of elders with dementia [12]. It can also be effective in other samples facing stress or adversity. This study provides evidence for a psychometrically sound measure to directly evaluate the teaching of specific personal and social resourcefulness skills in family members of persons with serious mental illness. However, to enhance the usefulness of the scale, further psychometric testing with other populations is needed. 

There were a number of limitations to the study. First, this was a secondary analysis, and while there were sufficient data to perform the psychometric analysis, we were limited in the validation measures available to us. Second, the use of convenience sampling limits the generalizability of the findings. Third, the data were obtained through self-report, though it should be noted that the perspective of the intervention recipient is needed and this may justify the use of self report. Fourth, it is possible that study participants may have responded to questions on the RSS in a socially desirable manner. Although the development and testing of an observational scale to evaluate use of resourcefulness skills would provide more objective data, such information may not reflect the perspective of the intervention recipient. Finally, the sample size was modest though it exceeded the minimum requirement of four subjects per item on a scale recommended for psychometric analysis [28]. The subject to item ratio for the 8-item RSS was 1 to 7.5. Despite these limitations, the findings from the current study provide promising evidence of the reliability and construct validity of the Resourcefulness Skills Scale, which has the potential for providing an intervention-specific measure to assess the fidelity of resourcefulness training.

## Figures and Tables

**Table 1:** Descriptive statistics for the Resourcefulness Skills Scale and construct validation measures in women family members (*N* = 60).

Validating construct	Mean (St. Dev.)	Possible range	Cronbach's alpha
Learned resourcefulness	113.85 (18.08)	0–180	.81
Resourcefulness	89.83 (15.10)	0–140	.82
Depressive cognitions	8.37 (6.83)	0–40	.87
Quality of life	18.17 (8.16)	0–36	.91

**Table 2:** Reliability analysis with alphas if item deleted and corrected item-to-total scale correlations for the 8-item RSS in women family members (*N* = 60).

RSS item	Alpha if deleted	Item-to-total scale correlation
Rely on family/friends	.763	.446
Exchange ideas with others	.768	.413
Seek professionals/experts	.764	.456
Organize daily activities	.760	.463
Use positive self-talk	.757	.481
Reframe the situation positively	.742	.575
Change from usual reaction	.732	.633
Explore new ideas	.766	.425

**Table 3:** Interitem correlations among the 8 RSS items in women family members of adults with serious mental illness (*N* = 60).

RSS item	2	3	4	5	6	7	8
(1) Rely on family/friends	.438	.406	.189	.266	.301	.277	.111
(2) Exchange ideas with others		.543	.164	.058	.211	.370	.001
(3) Seek professionals/experts			.178	.164	.215	.420	.116
(4) Organize daily activities				.438	.404	.259	.499
(5) Use positive self-talk					.555	.402	.307
(6) Reframe the situation positively						.495	.393
(7) Change from usual reaction							.370
(8) Explore new ideas							

**Table 4:** Exploratory factor analysis of RSS: two factors emerged.

RSS	Two-factor solution factor loadings	
Rely on family/friends		.510
Exchange ideas with others		.849
Seek professionals/experts		.692
Organize daily activities	.605	
Use positive self-talk	.655	
Reframe the situation positively	.672	
Change from usual reaction	.538	
Explore new ideas	.714	
